# Generation of A *Mucor circinelloides* Reporter Strain—A Promising New Tool to Study Antifungal Drug Efficacy and Mucormycosis

**DOI:** 10.3390/genes9120613

**Published:** 2018-12-07

**Authors:** Ulrike Binder, Maria Isabel Navarro-Mendoza, Verena Naschberger, Ingo Bauer, Francisco E. Nicolas, Johannes D. Pallua, Cornelia Lass-Flörl, Victoriano Garre

**Affiliations:** 1 Division of Hygiene and Medical Microbiology, Medical University Innsbruck, Schöpfstrasse 41, 6020 Innsbruck, Austria; verena.naschberger@i-med.ac.at (V.N.); cornelia.lass-floerl@i-med.ac.at (C.L.-F.); 2 Departamento de Genética y Microbiología, Facultad de Biología, Universidad de Murcia, 30100 Murcia, Spain; mariaisabel.navarro3@um.es (M.I.N.-M.); fnicolas@um.es (F.E.N.); 3 Division of Molecular Biology, Biocenter, Medical University of Innsbruck, Innrain 80-82, 6020 Innsbruck, Austria; ingo.bauer@i-med.ac.at; 4 Institute of Pathology, Neuropathology and Molecular Pathology, Medical University of Innsbruck, Müllerstraße 44, 6020 Innsbruck, Austria; johannes.pallua@i-med.ac.at

**Keywords:** *Mucor circinelloides*, mucormycosis, firefly luciferase, reporter strain, bioluminescence

## Abstract

Invasive fungal infections caused by Mucorales (mucormycosis) have increased worldwide. These life-threatening infections affect mainly, but not exclusively, immunocompromised patients, and are characterized by rapid progression, severe tissue damage and an unacceptably high rate of mortality. Still, little is known about this disease and its successful therapy. New tools to understand mucormycosis and a screening method for novel antimycotics are required. Bioluminescent imaging is a powerful tool for in vitro and in vivo approaches. Hence, the objective of this work was to generate and functionally analyze bioluminescent reporter strains of *Mucor circinelloides*, one mucormycosis-causing pathogen. Reporter strains were constructed by targeted integration of the firefly luciferase gene under control of the *M. circinelloides* promoter P*zrt1*. The luciferase gene was sufficiently expressed, and light emission was detected under several conditions. Phenotypic characteristics, virulence potential and antifungal susceptibility were indifferent to the wild-type strains. Light intensity was dependent on growth conditions and biomass, being suitable to determine antifungal efficacy in vitro. This work describes for the first time the generation of reporter strains in a basal fungus that will allow real-time, non-invasive infection monitoring in insect and murine models, and the testing of antifungal efficacy by means other than survival.

## 1. Introduction

*Mucor circinelloides*, a member of the Mucoromycota, is ubiquitously found in the environment. It is thermotolerant, able to grow on a wide range of organic substrates and sporulates fast and abundantly [1,2]. It can cause mucormycosis—a severe animal and human disease. In recent decades, the incidence of mucormycosis has increased all over the world, becoming the second most common fungal disease in patients with haematological malignancies and transplant recipients [3,4,5]. Infections with mucormycetes are highly aggressive and destructive, resulting in tissue necrosis, invasion of blood vessels and subsequent thrombosis. The rapid progression, linked with shortcomings in diagnosis and therapy, results in high mortality rates which are estimated to range between 40–>90%, depending on the site of infection, the condition of the host and the therapeutic interventions [3,5,6,7]. The different types of mucormycosis are classified according to the anatomic site of infection, such as rhino-orbital-cerebral, pulmonary, cutaneous, gastrointestinal and disseminated infections [8]. Antifungal therapy is complicated by the limited treatment options that comprise lipid amphotericin B (AMB) as first-line therapeutic and posaconazole (POS) or isavuconazole (ISA) as salvage treatment [9,10]. 

The most common genera associated with human disease next to *Mucor* are *Rhizopus*, and *Lichtheimia* (formerly *Absidia*) and infections are associated with severe graft-versus-host disease, treatment with steroids, neutropenia, iron overload, diabetes and malnutrition [10]. *M. circinelloides* isolates have been associated with outbreaks of mucormycosis in the US, the UK and Europe and it poses a threat to public health by contaminating food and producing 3-nitropropionic acid [11,12,13,14,15]. 

Despite the growing relevance of mucormycetes in public health, little is known about the physiology and virulence factors associated with this group of fungi. The heterogeneity of this group and the difficulties in genetic manipulation are reasons thereof. However, *M. circinelloides* stands out among the rest of basal fungi offering the opportunity to carry out genetic manipulation by the development of an increasing number of molecular tools [16,17]. The intrinsic resistance of mucormycetes to drugs used as resistant markers in other fungi, leaves only the use of auxotrophic markers [18,19].

Bioluminescence imaging is a very useful technique to track microorganisms in living animals and has provided novel insights into the onset and progression of disease. The great advantage is the real-time monitoring of infection in one individual organism over time. Different enzymes exist in living organisms, which use different substrates and different cofactors to emit light. The most prominently used are the firefly (*Photinus pyralis*) and the copepod (*Gaussia princeps*) luciferase [20]. Both have already been successfully transformed into opportunistic fungal pathogens e.g., *Candida albicans* [21,22], *Aspergillus fumigatus* [23,24,25] and *A. terreus* [26]. Bioluminescence imaging with these strains has significantly enhanced our understanding of fungal infection. It revealed unexpected host sites in disseminated candidiasis, showing persistence of *Candida* cells in the gallbladder, even after antifungal treatment. Studies comparing *A. fumigatus* and *A. terreus* revealed delayed onset of disease in *A. terreus* infected mice and survival in 50% of *A. terreus* infected mice, although progression of disease was similar to those that died. 

In this study, we generated bioluminescent strains in the opportunistic human pathogen *M. circinelloides* based on the expression of firefly luciferase and controlled under a highly expressed *M. circinelloides* promoter, for the first time. Light emission was correlated to fungal growth and concentration of the substrate luciferin. Phenotypic analysis and virulence potential in the alternative host *Galleria mellonella* revealed no differences to parental strains. Antifungal efficacy was determined successfully by the use of the obtained reporter strains. The strains generated in this study will be a useful tool to test novel antifungal agents both in vitro and in murine and insect models, in addition to shed light on the onset and progression of mucormycosis in animal models.

## 2. Material and Methods

### 2.1. Fungal Strains, Plasmids, Media and Growth Conditions

The strains and plasmids used in this study are listed in Table 1. All fungal strains used were *M. circinelloides f. lusitanicus*, referred to in this work as *M. circinelloides* for simplicity. To obtain spores, strains were grown on YPG (yeast peptone glucose agar; 3 g/L yeast extract, 10 g/L peptone, 20 g/L glucose, pH 4.5) medium at 26 °C in the light for 4–5 days. Spores were collected by scraping the plates with sterile spore solution buffer (0.9% NaCl, 0.01% Tween 80) and spore concentration was determined by hemocytometer. Media and growth conditions for the individual assays are given below, for most assays YNB (yeast nitrogen base; 1.5 g/L ammonium sulfate, 1.5 g/L glutamic acid, 0.5 g/L yeast nitrogen base (*w*/*o* ammonium sulfate and amino acids, Sigma-Aldrich, Steinheim, Germany, cat. no. Y1251), 10 g/L glucose, thiamine 1 µg/mL and niacin 1 µg/mL) was used [16]. All chemicals used were purchased from Sigma-Aldrich, Germany, unless otherwise stated.

*Escherichia coli* DH5α was used as a host for plasmid propagation and grown in lysogeny broth (LB) medium, supplemented with 0.1 mg/mL ampicillin if needed. Bacterial cultures were incubated at 37 °C overnight.

### 2.2. Cloning Procedures

#### 2.2.1. Amplification of the Firefly Luciferase Gene

The plasmid pGL3 basic vector (Promega, Fitchburg, WI, USA) served as a template to amplify firefly luciferase by PCR using primers luc-FOWXhoI (AAACTCGAGATGGAAGACGCCAAAAACATAAAGAAAGG), luc-REVSacII (CGCCCCGCGGCTAGAATTACACGGCGATCTTTCC) and Herculase II fusion DNA polymerase (Agilent, Santa Clara, CA, UAS). This luciferase is an optimized version for the use in mammalian cells and does not contain the peroxisomal target sequence of the native firefly luciferase. 

#### 2.2.2. Plasmid Construction 

The pMAT1477 plasmid carries the strong promoter of the *M. circinelloides zrt1* gene (P*zrt1*) and a functional *leuA* gene as a selective marker [19]. Amplified luciferase gene was digested by *Xho*I and *Sac*II and ligated into pMAT1477 to obtain pMAT1903 (Figure S1). By the targeted integration of the whole construct in the *M. circinelloides carRP* gene, which is involved in carotenoid biosynthesis, identification of clones with integrated gene was facilitated, as they formed albino colonies, while those without integration had a yellow phenotype [28].

### 2.3. Transformation of *Mucor circinelloides* and Initial Screening

Strain R7B (*leuA^−^*) and MU402 (*leuA^−^, pyrG^−^*) were chosen as recipient strains. MU402 is derived from R7B. Transformation of *M. circinelloides* was performed by electroporation of protoplasts as described previously [16]. In brief, freshly harvested spores were incubated in YPG media (pH 4.5) for 2–4 h until spores were germinated and then transferred to a fresh tube for digestion of the cell wall by lysing enzymes (L-1412, Sigma-Aldrich, St. Louis, MO, USA) and chitosanase (C-0794, Sigma-Aldrich). Linearized DNA (5 µg) was used in each transformation reaction. Protoplasts were incubated on YNBS agar (pH 3.2; containing 0.2 g/L uridine for MU402 strains) and checked daily for colonies [16]. Colonies formed by protoplasts with correct gene targeting appeared white, because of disruption of the *carRP* gene by targeted integration. Albino colonies were repeatedly transferred to fresh selective agar plates (3–4 cycles) to obtain homokaryons. Several clones in each background were then chosen and tested for light emission. Therefore, spores were incubated in YPG in 6-well plates (Nunc GmbH, Langenselbold, Germany) overnight, then D-luciferin (10 mM, Synchem, Felsberg, Germany) was added to the cultures and light emission detected by a monochrome scientific grade CCD camera (BIO-Vision 3000 imaging system, Golden, CA, USA (Figure S3)). Clones that showed highest light emission were chosen for further experiments.

### 2.4. Genomic DNA Extraction and Southern Analysis

For the preparation of genomic DNA, lyophilized mycelia were ground to powder using a tungsten carbide ball in a Retsch MixerMill 400 and resuspended in 1 mL of DNA isolation buffer (50 mM Tris-HCl, 250 mM NaCl, 100 mM EDTA (Ethylenediaminetetraacetic acid), 1% (*w*/*v*) SDS (Sodium dodecyl sulfate), pH 8.0 at 25 °C) and 300 µL of PCI (Phenyl-Chloroform-Isoamylalcohol; Carl Roth, Karlsruhe, Germany). After incubation at room temperature for 5 min, the mixture was centrifuged for 10 min at 20,000× *g* and 4 °C. RNase A (10 µL; 10 mg/mL) were added to the supernatant and incubated for 10 min at 65 °C and further 30 min at 37 °C. After RNase digestion, DNA was extracted by addition of 1/3 volume of PCI, centrifuged and the resulting supernatant was precipitated by addition of 1 volume of isopropanol. The DNA pellet was washed with 180 µL of 70% ethanol, briefly air-dried and solubilized in 50 µL of a.d. Concentration and quality were determined by agarose gel electrophoresis. 2 µg of genomic DNA were digested overnight with 10–20 units of either *Bgl*II or *Pst*I and separated on 0.8% agarose gels. DIG-labeled marker VII (Roche, Basel, Switzerland) served as a marker for fragment size estimation. Capillary transfer of DNA onto nylon membrane was performed overnight. Membranes were hybridized with a probe for the luciferase coding sequence. Probe labeling with DIG-dUTP was performed by PCR amplification using primers luc1f (5′-TCGCATGCCAGAGATCCT) and luc1r (5′-CGCCCGGTTTATCATCCC).

### 2.5. Luciferase Activity

Luciferase activity was tested in vitro by measuring light emission of bioluminescent *M. circinelloides* strains in dependency of inoculum density and growth conditions by luminometer Tecan infinite 200 PRO plate reader (Tecan Group AG, Männedorf, Switzerland). First, two transformants of each background were chosen, grown in YNB (2 × 10^5^ spores/mL) in 24-well microtiter plates (Nunc) for 24 h, 100 µL of luciferin (Roche, Luciferase Reporter Gene Assay) were added to the cultures and light emission was detected immediately (2 min after addition of luciferase), or 30 min after addition of substrate to check for stability of light emission. 

To determine correlation to inoculum size and subsequent fungal growth/biomass, different spore concentrations were incubated (2 × 10^2^–2 × 10^6^/mL) and light emission was detected as previously described. 

A dilution series of substrate luciferin (Roche)—undiluted, 1:2, 1:5 and 1:10—was tested to check for the optimal concentration needed for further experiments. 

To determine if light emission is dependent upon growth conditions, strains (2 × 10^5^ spores/mL) were pre-grown in YNB to the same amount of biomass overnight, before the medium was replaced by fresh YNB, YPG or RPMI_1640_ (Sigma-Aldrich, Spittal/Drau, Austria), respectively. Substrate addition and measurement of emitted light were carried out as described above.

### 2.6. Phenotypic Analysis in Different Growth Conditions

Growth on different media of the recipient strains and the resulting luciferase expressing clone was compared on different media (YNB, YPG, RPMI_1640_ and supplemented minimal agar, SUP [29]; with supplements added when necessary). 10^4^ spores of the individual strains were dotted onto the respective agar plates and incubated at 30 °C and 37 °C, respectively. After 24 h colony diameters were measured and growth documented visually. Experiments were carried out with 3 parallels and repeated twice. For growth assays in hypoxic conditions cultures were grown at 1% O_2_ (Biospherix, C-Chamber & Pro-Ox controller, Parish, NY, USA).

### 2.7. Antifungal Susceptibility Testing

Minimum inhibitory concentration (MIC) of AMB, POS, ISA, and itraconazole (ITRA) were determined for all strains according to the European Committee on Antimicrobial Susceptibility Testing (EUCAST) guidelines 9.2 [30]. MIC was defined as the lowest concentration that completely inhibited growth. Additionally, MICs were also determined in YNB medium, as this was used for luciferin activity assays. 

To determine antifungal drug efficacy by correlation of fungal growth with light emission, R7B_luc was grown in YNB overnight in the presence of AMB (0.25 µg/mL and 1 µg/mL) or POS (2, 16 and 32 µg/mL), respectively, in 96 well plates. To test the growth inhibiting activity of AMB and POS on *M. circinelloides* hyphae, cultures were pre-grown in YNB overnight before antifungal drugs were added. After 4 h of drug exposure, luciferin was added and light emission detected as described above. All experiments were carried out in parallels and repeated twice.

### 2.8. Virulence Assay in *Galleria mellonella*

Sixth instar larvae of *G. mellonella* (SAGIP, Bagnacavallo, Italy), weighing 0.3–0.4 g, were selected for experimental use. Larvae, in groups of twenty, were injected through the last pro-leg into the hemocoel with 1 × 10^6^ spores in a volume of 20 µL as described previously and incubated at 30 °C in the dark [29,31,32]. Untouched larvae and larvae injected with sterile insect physiological saline (IPS) served as controls. Survival was determined every 24 h over a period of 144 h. Experiments were repeated three times and the average survival rate was calculated. Significance was determined with log-rank (Mantel-Cox) test, utilizing GraphPad Prism 7 software (GraphPad Software, San Diego, CA, USA). Differences were considered significant at *p*-values < 0.05.

### 2.9. Histology of Larvae

Specimen were fixed in formalin for at least 15 days before being embedded in paraffin. Longitudinal tissue sections were carried out with a microtome at 3.0 µm thickness and stained with Grocott for histological validation. Slides were digitalized using a Pannoramic SCAN digital slide scanner (3DHISTECH, Budapest, Hungary) with plan-apochromat objective (magnification: 20×, Numerical aperture: 0.8). The histological evaluation and the scoring of the fungal infection were done by using the Pannoramic Viewer software (3DHISTECH). 

## 3. Results and Discussion 

### 3.1. Generation of Firefly Luciferase-Producing *Mucor circinelloides* Strains Resulted in Detectable Light Emission

For the generation of luciferase-producing *M. circinelloides* strains, we cloned the firefly (*P. pyralis*) luciferase gene, optimized for use in mammalian cells, under the control of a strong *M. circinelloides zrt1* promoter into plasmid pMAT1477 that contained the *leuA* gene, which was used as a selective marker in transformations. In the resulting plasmid, the luciferase gene and *leuA* are flanked by sequences of the *carRP* locus to favor targeted integration of the whole construct. The plasmid was linearized and then used to transform the leucine auxotroph strain R7B and the leucine and uridine auxotroph strain MU402. The double auxotroph was chosen to facilitate subsequent disruption or introduction of other genes in the bioluminescent strain. In both backgrounds, more than 50 colonies were obtained on selective transformation plates. Integration in the *carRP* locus renders albino colonies, hence fifteen independent transformants of each background with white appearance were selected. Due to the multinucleated nature of the protoplasts, they were repeatedly inoculated on selective agar until the transformants produced only white colonies, an indication that they were homokaryons. Five of these transformants per background were randomly selected and checked for luciferase production by observing light emission with the naked eye in the dark and visualization of light production (Figure S3). Two strains in each background, showing high light emission, were selected for Southern blot analysis using digoxigenin-labeled probes directed against the luciferase coding region (Figure S2). Restriction with *Bgl*II or *Pst*I confirmed correct insertion and single integration of the luciferase gene. The same four strains were chosen for further luminescence detection by microplate reader and CCD camera (Figure S3). As shown in Table 2, all transformants emitted more light than the parental strains, indicating that our approach of expressing firefly luciferase for the first time in *M. circinelloides* was successful. Measurements taken 30 min after substrate addition indicated that light emission was moderately stable and still significantly detectable after this time, which is essential for further use of the reporter strains. Furthermore, luciferase-harboring strains were stable over several generations because of the site- directed insertion and the selection for homokaryons. Light signals were lower for transformants in the MU402 background, which correlates to slower growth and less biomass of these strains compared to R7B. Therefore, mainly R7B_luc was used for further experiments. Regarding the difficulties with genetic engineering of basal filamentous fungi and in particular the opposition of mucormycetes to express foreign genes, this is an achievement that will be advantageous for further experimental work (e.g., in optimizing luciferase expression in *M. circinelloides* and other mucormycetes).

### 3.2. In Vitro Characterization of Bioluminescent *Mucor circinelloides* Reporter Strains 

#### 3.2.1. Radial Growth Is Not Altered by Insertion of the Luciferase Gene

**Figure 1:**
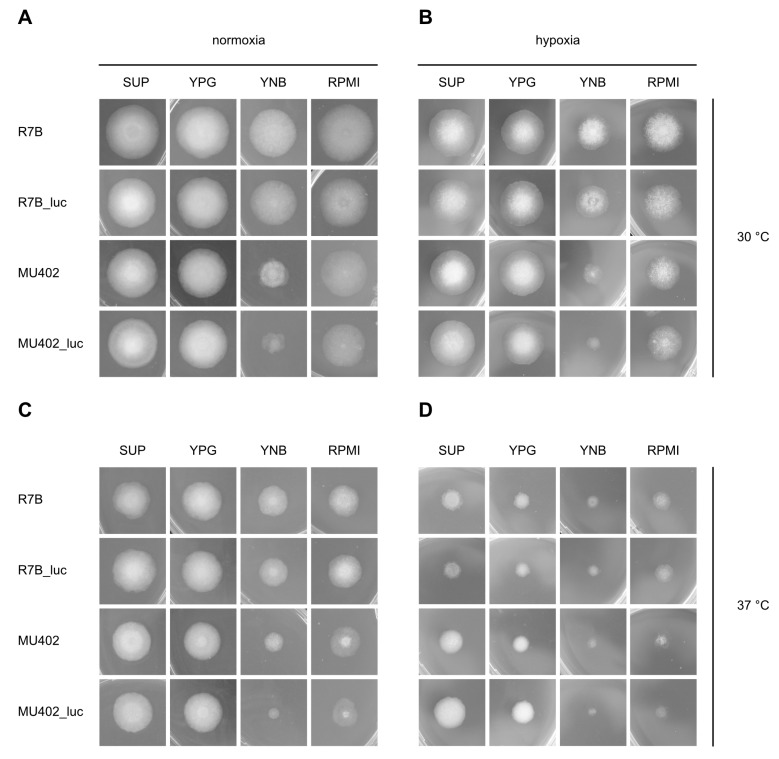
Growth phenotypes of recipient and luciferase-expressing strains grown for 24 h on different media at 30 °C (panels (**A**,**B**)), 37 °C (panels (**C**,**D**)) under normoxic (panels (**A**,**C**)) and hypoxic conditions (panels (**B**,**D**)). Hypoxia was induced by reducing the oxygen concentration in the incubator to 1%. SUP: supplemented minimal agar; YPG: yeast peptone glucose; YNB: yeast nitrogen base; RPMI: RPMI_1640_.

To check growth ability of luciferase containing strains compared to their recipient strains, radial growth was determined on different media at 30 °C and 37 °C. Based on results shown in Table 2, R7B_luc and MU402_luc were chosen for the radial growth assays, because they showed higher light emission. R7B_luc is prototrophic, while MU402_luc is still auxotrophic for uridine. The aim to generate a reporter strain in the MU402 background was to have a tool in hand that can be used for further genetic manipulation, such as deletion of genes essential for virulence. Here, to rule out phenotypes resulting from luciferase integration, also the MU402_luc strains were used for growth characterization and light emission assays. However, these strains will not be used per se in future animal models. None of the strains displayed an obvious abnormal growth phenotype. At both temperatures, R7B_luc exhibits same growth and average colony diameter on each of the media tested compared to the parental strain R7B (Figure 1, Table S1). MU402_luc exhibited significantly smaller colony diameters on YNB (containing uridine) at both temperatures and on RPMI_1640_ at 37 °C, but still, no differences were detected compared to the parental strain. As expected, colonies were smaller at 37 °C at this early time point, indicating difficulties of *M. circinelloides* with adaptation to high temperatures. Because oxygen levels are expected to be very low on site of infection in the human and animal body [33], and we aim to use the luciferase containing strains in animal models, growth was further evaluated in hypoxic conditions (1% oxygen). Even at this low oxygen concentration, all strains were able to grow and form hyphae, a pre-requisite of tissue invasion. Growth was reduced in all samples compared to normoxic conditions, especially on minimal media (YNB and RPMI). Surprisingly, MU402 and MU402_luc seemed to adapt better to the combination of low oxygen and elevated temperature than R7B_luc and its parental strain on SUP and YPG. At 48 h growth in hypoxia was restored at 30 °C and partially at 37 °C (Figure S4), indicating that *M. circinelloides* spores showed delayed germination in hypoxic conditions, but are able to adapt to low oxygen. This ensures that the luciferase strains will be suitable for use in infection models at a later time point.

#### 3.2.2. Light Emission Correlates with Fungal Biomass and Amount of Available Luciferase Substrate

**Figure 2:**
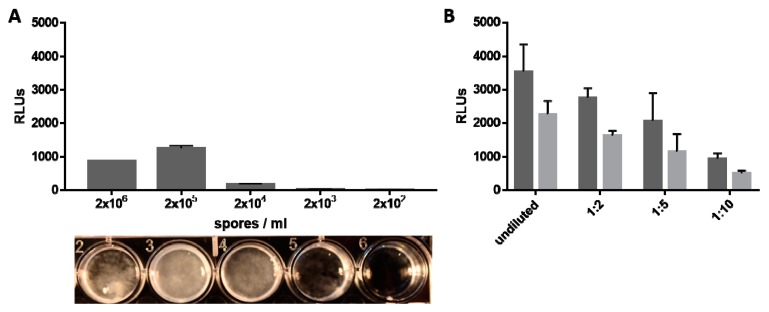
(**A**) Light emission in dependency of inoculum density. YNB medium was inoculated with different concentrations of R7B_luc spores, light emission was induced by addition of luciferin after 24 h and detected by plate reader (upper panel). Relative light units (RLUs) represent the average of three independent measurements. Error bars indicate standard deviation. The lower panel shows fungal growth at the various spore concentrations after 24 h of incubation. (**B**) Light emission in dependency of substrate concentration. 2 × 10^5^ spores/mL were inoculated in YNB and light emission was induced by addition of different concentrations (undiluted, 1:2, 1:5; 1:10) of luciferin dissolved according to the manufacturer´s protocol (Roche, Basel, Switzerland). Light was detected immediately (dark grey bars) and 10 min after substrate addition (light grey bars). Error bars indicate standard deviation.

An important parameter for the use of bioluminescent reporter strains in animal infection is the detection limit of emitted light, which was determined by cultivation of R7B_luc spores at different inoculum densities and assessment of light emission after 24 h of growth. Light was detected in cultures inoculated with as low as 2 × 10^3^ spores/mL compared to the controls without luciferin and increased with the number of spores used. Highest RLUs were observed at a spore concentration of 2 × 10^5^/mL (Figure 2A, upper panel), the spore concentration that also led to the highest density of mycelia (Figure 2A, lower panel). All other spore concentrations led to significantly lower RLU measurement (*p* < 0.05). The highest inoculum concentration used (2 × 10^6^/mL) did not result in highest light emission, which can be explained by lower growth rate and non-homogeneity of in the culture. Spores probably germinate but face a lack of nutrients at this high density. For all further in vitro experiments, 2 × 10^5^ spores were used.

Different concentrations of the substrate luciferin were tested to evaluate the minimum amount necessary for R7B_luc to emit detectable light. As expected, light emission clearly correlated with amount of substrate added to the cultures (Figure 2B). As shown before, light emission decreased with time, with significantly reduced light emission at the later time point (*t*-test, *p* < 0.05), but was still detectable 10 min after substrate addition.

**Figure 3:**
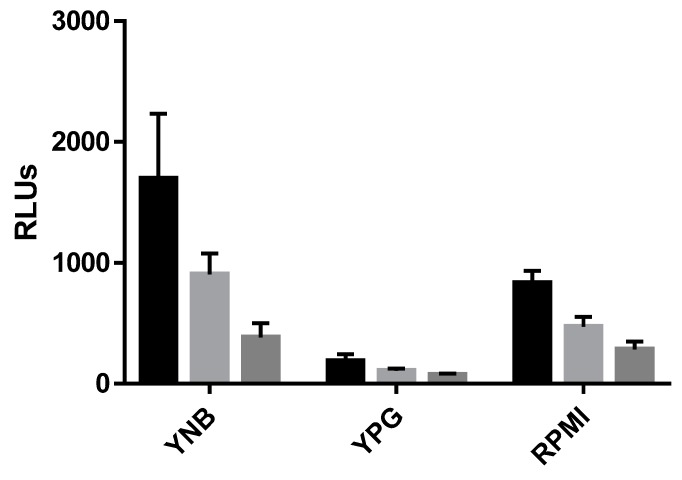
Light emission of R7B_luc in different growth media. YNB medium was inoculated with 2 × 10^5^ spores/mL of R7B_luc, incubated for 16 h, and then replaced by fresh YNB, YPG, or RPMI, respectively. Light emission was induced by addition of substrate (luciferin 1:5) 3 h after medium exchange and detected by using a plate reader. RLUs were determined immediately after addition of substrate (black bars), 10 min (light grey bars) and 30 min (dark grey bars) after the addition of substrate. Error bars indicate standard deviation. RLUs emitted in each media were significantly different from the other media tested (Two-way analysis of variance (ANOVA), *p* < 0.05).

To evaluate the effect of growth media on luciferase expression, cultures were pre-grown in YNB overnight and the medium was replaced by fresh YNB, YPG or RPMI_1640_ 3 h before addition of substrate and light detection. Measurement of RLUs revealed highest levels of emitted light in YNB medium and lowest in YPG (Figure 3). One possible explanation is the nature of the P*zrt1* promoter used for our construct. The gene *zrt1* codes for a zinc transporter whose expression is induced by reduced availability of zinc as is the case in minimal media such as YNB. In mammalian tissues such as human lung or blood, the concentration of zinc is very low; therefore, expression of luciferase driven by P*zrt1* should be high in vivo. Rich media, such as YPG contain higher concentrations of zinc, hypothetically resulting in downregulation of luciferase expression.

### 3.3. Antifungal Susceptibility Testing

#### 3.3.1. Genetic Manipulation Does Not Influence Antifungal Susceptibility Patterns of *Mucor circinelloides* Strains

The expression of the luciferase gene does not affect the susceptibility patterns of *M. circinelloides* to commonly used antifungal agents, since strains expressing the luciferase gene showed the same susceptibility pattern as the recipient strains (Table 3). To better compare MIC results with results from light emission studies, MICs were also determined in YNB in addition to RPMI_1640_ medium. All strains showed moderate susceptibility to AMB, resulting in complete growth inhibition at concentrations between 0.5 and 2 µg/mL in both media tested. This correlates well to other studies, and thus all 4 strains tested could be classified as susceptible to AMB according to the epidemiological cut-off value determined for *M. circinelloides* [34]. Despite of reducing growth, the azole concentrations applied were below the MIC in RPMI_1640_, nevertheless, in YNB a posaconazole MIC could be determined for R7B and R7B_luc. Although POS is regarded as second-line treatment, it has been shown before that *M. circinelloides* isolates very often also exhibited resistance to this azole [35,36]. The fact that susceptibility patterns of luciferase-harboring and recipient strains were very similar, assures that the luciferase-harboring strains are suitable for the assessment of antifungal drug efficacy in vitro and in vivo. 

#### 3.3.2. Bioluminescent Strains Can Be Used to Evaluate Efficacy of Antifungal Drugs

**Figure 4:**
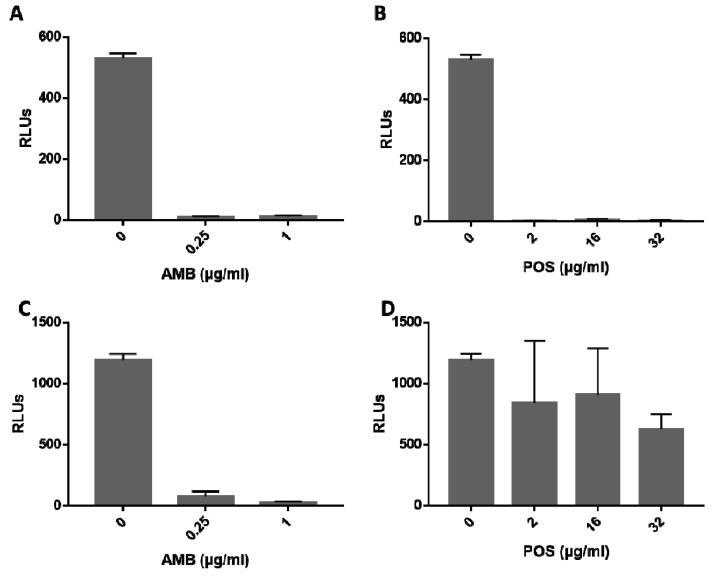
Graphical analysis of drug efficacy by detection of light emission. Luminescence was measured by a plate reader in wells containing 2 × 10^5^ spores/mL of R7B_luc grown in YNB in the presence of amphotericin B (AMB) (**A**) or posaconazole (POS) (**B**), and with AMB (**C**) or POS (**D**) added to hyphae, respectively. Light emission was detected after 24 h of incubation (**A**,**B**) and subsequent luciferin (1:5, Roche) addition. Pre-grown cultures (16 h) were further incubated for 4 h once antifungals were added (**C**,**D**). Average values from three independent wells are given, error bars represent standard deviation.

To test if the luciferase producing strain R7B_luc is suitable for monitoring the efficacy of antifungal substances, we used AMB and POS in two different experimental setups. First, the respective antifungal agent was added directly to spores of R7B_luc, mimicking the EUCAST protocol and light emission was determined after 24h of incubation. No light was detected in wells containing either AMB or POS; respectively (Figure 4A,B). Results obtained with 1 µg/mL AMB correlate well with the MIC determined (Table 3). At this concentration no growth was evident in the presence of AMB prior to germination and consequently no light is emitted. Although some spores could grow in the presence of 0.25 µg/mL AMB, also at this concentration biomass was too little to produce sufficient luciferase. Similarly, of all three POS concentrations tested, none resulted in the emission of detectable light units. Regarding a MIC of 4 mg/mL determined in YNB medium—the same medium that was used for the light emission assays—evidence of a correlation to standard MIC testing is observed. Even at a concentration of 2 µg/mL, growth (or at least fungal metabolism) was inhibited sufficiently to prevent production of luciferase and consequently, light.

The second approach was to test to what extent the production of luciferase is affected by the addition of antifungal agent, AMB and POS, to R7B_luc hyphae. AMB showed a tremendous effect on luciferase activity, resulting in no light emission when hyphae were incubated in 1 µg/mL AMB for 4 h, and only marginal light emission at 0.25 µg/mL (Figure 4C). This suggests a strong effect of AMB on hyphal metabolism, presumably resulting in decreased ATP levels within the hyphae, which consequently leads to reduced activity of the ATP-dependent luciferase. Another possible effect could be inhibition of substrate uptake due to AMB-induced metabolic changes or membrane dysfunction. Hyphae confronted with POS for 4 h exhibited reduced light emission, but with only a significant difference at 32 µg/mL compared to the untreated controls (Figure 4D). This correlated to previous data that showed reduced effect of POS on hyphae compared to AMB [37]. Furthermore, poor in vitro and in vivo POS efficacy against several *M. circinelloides* strains, due to a rather fungistatic than a fungicidal activity of POS, was shown by Salas et al. [38]. The results obtained with the luciferase-producing strains are in agreement with the MIC data shown before and studies undertaken by others [34,35]. Therefore, we can conclude that screening for antifungal drug efficacy is possible by using luciferase-expressing *M. circinelloides* strains and presents a valuable tool for testing novel antifungal drugs. For *M. circinelloides*, or Mucorales in general, this is of great importance, because many laboratories face difficulties applying and interpreting standard susceptibility test procedures, such as microbroth dilutions methods (EUCAST or clinical and laboratory standards institute (CLSI)) and especially Etest^®^, with this group of fungi. Often, results obtained with different methods do not correlate [39,40,41]; therefore, the use of bioluminescent strains will be an additional possibility to test the efficacy of (novel) antifungal drugs or combinations thereof.

### 3.4. Luciferase-Harboring Strains Exhibit Similar Virulence Potential as Recipient Strains in the Alternative Host *Galleria mellonella*

**Figure 5:**
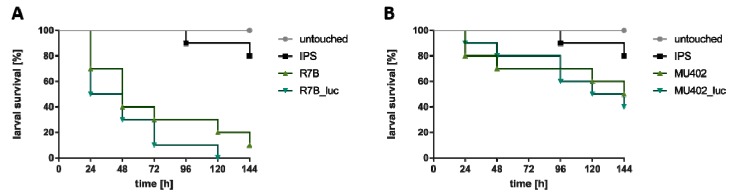
Survival of larvae infected with *M. circinelloides strains.* Larvae were infected with 10^6^ spores of the respective strains and incubated at 30 °C. (**A**) represents Kaplan-Meier curves of larvae infected with R7B or R7B_luc strain. (**B**) represents Kaplan-Meier curves of larvae infected with MU402 or MU402_luc strain. Survival was monitored every 24 h up to 144 h. Untouched larvae and larvae injected with IPS buffer served as controls. Results are expressed as the mean of three independent experiments (60 larvae in total).

In order to test whether the integration of the luciferase gene influenced the virulence potential of the *M. circinelloides* strains, infection studies in the invertebrate host model *G. mellonella* were carried out. All strains were able to cause death to the larvae and no significant difference (*p* > 0.05) was detected between the luciferase-containing strains and the recipient strains (Figure 5). Lower mortality rates seen for MU402 and MU402_luc are most likely due to uridine auxotrophy, suggesting limited availability of uridine or uracil in *Galleria* hemolymph. This correlated to data obtained with *A. fumigatus*, that showed attenuated virulence potential of uridine or uracil auxotrophic strains in murine models [42]. Ability to cause disease in *Galleria* larvae and similarities in survival rates of luciferase-harboring and recipient strains confirms suitability of generated strains for future use in in vivo models. Fungal elements were found in tissue sections of larvae infected with R7B_luc (Figure S5), indicating larval killing by active fungal growth within the larval body. This is important for further studies, in which we aim to use this model system for in vivo bioluminescent imaging.

For other fungi, such as *A. fumigatus* and *C. albicans*, codon-optimized luciferase gene sequences were used and resulted in increased light emission, successfully detected in murine models [22,23,43]. Codon optimization of luciferase in *M. circinelloides* would probably increase luciferase expression and result in higher levels of detectable light, which would specifically be important in murine models, as light detection is quenched by tissue. Further, correlation between the copy number of luciferase and light emission was shown in *A. terreus* and *A. fumigatus* strains [23,26]. Although *M. circinelloides* is one of the few genetically tractable species among the basal fungi, it is not yet a robust genetic system, such as *A. nidulans* or *A. fumigatus*, and knowledge of other native, strong promoters besides *zrt1* is scarce. Nevertheless, using alternative promoters and/or integration of additional luciferase gene copies could further improve our model system. 

## 4. Conclusions

The construction of bioluminescent reporter strains in the basal fungus *M. circinelloides* was successful and resulted in the first *M. circinelloides* strains expressing firefly luciferase, evident by detectable light emission. When comparing our newly generated reporter strains with their respective recipients, we obtained similar results regarding growth, antifungal susceptibility patterns, and virulence potential in the insect model *G. mellonella.* Further, luciferase-containing strains proved to be suitable for the evaluation of antifungal agents. The reporter strains obtained in this study represent a valuable tool for studies investigating the efficacy of novel antifungal agents and monitoring disease in a spatial and temporal manner in animal models in the future.

## Figures and Tables

**Table 1:** List of strains and plasmids used in this study.

	Name	Genotypes/Characteristics	Reference
*Mucor circinelloides*	R7B	*leuA1* (mutant alelle of *leuA* gene)	Roncero et al., 1984 [27]
	R7B_luc	*carRP*::*leu*	Obtained in this study
	R7B_luc1	*carRP*::*leu*	Obtained in this study
	MU402	*leuA1*, *pyrG*^−^	Nicolas et al., 2007 [18]
	MU402_luc	*pyrG*^−^, *carRP*::*leuA*	Obtained in this study
	MU402_luc1	*pyrG*^−^, *carRP*::*leuA*	Obtained in this study
	DH5α	ampicillin resistance	Thermo Fisher (Germering, Germany)
*Escherichia coli*	pMAT1477	*Pzrt1*, *leuA*	Rodriguez-Frometa et al., 2013 [19]
	pGL3 basic vector	firefly luciferase reporter vector	Commercially available, Promega (Fitchburg, WI, USA)
*Plasmids*	pMAT1903	pMAT1477 + luciferase	Obtained in this study

**Table 2:** Detection of luminescence signal by microplate reader. 10^5^ spores/mL of the respective strains were grown in YNB medium (containing supplements where needed) for 24 h. Light emission was induced by the addition of D-luciferin (10 mM) and detected with a microplate reader (Tecan Group AG, Männedorf, Switzerland). Ten seconds were set as integration time. Measurements were carried out 2 min and 30 min after substrate addition. RLUs (relative light units) present the average of three experiments; SD represents standard deviation.

Strains	RLUs (2 min)	SD	RLUs (30 min)	SD
R7B	14	4	15	1
R7B_luc	4167	112	1444	129
R7B_luc1	3117	73	1194	95
MU402	10	1	7	3
MU402_luc	174	50	185	0
MU402_luc1	20	2	15	1

**Table 3:** Minimal inhibitory concentrations (MICs; µg/mL) determined for amphotericin B (AMB) and azoles (posaconozale: POS; itraconazole: ITRA; isavuconazole: ISA) according to European Committee on Antimicrobial Susceptibility Testing (EUCAST) guidelines. MICs were determined after 24 h of incubation at 37 °C, except for MU402 and MU402_luc, where MICs were read after 48 h of growth in yeast nitrogen base (YNB).

	MIC (µg/mL)							
	RPMI_1640_				YNB			
Strains	AMB	POS	ITRA	ISA	AMB	POS	ITRA	ISA
R7B	2	>16	>8	>4	1	2	>8	>4
R7B_luc	1	>16	>8	>4	0.5	4	>8	>4
MU402	2	>16	>8	>4	1	>16	>8	>4
MU402_luc	2	>16	>8	>4	1	>16	>8	>4

## Supplementary Materials

The following are available online at http://www.mdpi.com/2073-4425/9/12/613/s1, Figure S1: Plasmid pMAT1903 containing luciferase gene without peroxisomal target sequence under the strong zrt1 promoter and functional *leuA*, Figure S2: Gene targeting strategy and confirmation of homologous recombination. (A) Schematic presentation of gene targeting and Southern analysis strategy. For generation of the targeting cassette, a leucine auxotrophic marker followed by the gene coding for firefly luciferase were cloned in between 1 kb each of the 5’ UTR and the 3’ UTR of carRP, which were used as flanks for homologous recombination after linearization of the plasmid by *Cfr*9I (*Xma*I) digestion. Restriction sites (*Bgl*II, *Pst*I) and the site for probe hybridization are shown. Expected fragment length to be identified upon proper integration at the carRP locus are 4.6 kb and 5.3 kb. (B) Southern analysis. Genomic DNA of individual transformants was digested with *Bgl*II and *Pst*I, respectively, separated on a 0.8% agarose gel (lower panels) and blotted onto nylon membranes. To determine fragment size, DIG-labelled marker VII (Roche; M) was used; the length of selected marker bands is indicated, Figure S3: Visualisation of bioluminescence from *M. circinelloides* cultures. The *M. circinelloides* transformants (2 per strain) and the respective parental strains are shown. 105 spores/ml were inoculated in YNB medium and grown for 24 h. Light emission was induced by the addition of D-luciferin (10 mM) to the medium, and bioluminescence images of the cultures were acquired by a monochrome scientific grade CCD camera (BIO-VISION 3000 imaging system, right panel), Figure S4: Growth phenotypes of recipient and luciferase expressing strains grown for 48 h on different media at 30 °C (panels A and B) and 37 °C (panels C and D) under normoxic (panels A and C) and hypoxic conditions (panels B and D) are shown. Hypoxia was induced by reducing the oxygen concentration in the incubator to 1 %, Figure S5: Histological examination of *Mucor circinelloides* infected *Galleria mellonella* larvae. Specimen were fixed in formalin 72 h after infection with 106 spores of R7B_luc and embedded in paraffin. Tissue sections were prepared at a thickness of 3.0 μm and stained with Grocott silver stain to optimize visualisation of fungal elements, Table S1: Colony diameter of *M. circinelloides* strains on various growth media. Colony diameter was determined in triplicates after 24 h of incubation at 30 °C and 37 °C. Numbers given represent the average of two independent experiments. Significance was determined by calculating standard deviation (SD).

## References

[1] Richardson M. (2009). The ecology of the Zygomycetes and its impact on environmental exposure. Clin. Microbiol. Infect..

[2] Ingold C.T. (1978). The Biology of Mucor and Its Allies.

[3] Kontoyiannis D.P., Lionakis M.S., Lewis R.E., Chamilos G., Healy M., Perego C., Safdar A., Kantarjian H., Champlin R., Walsh T.J. (2005). Zygomycosis in a tertiary-care cancer center in the era of *Aspergillus*-active antifungal therapy: A case-control observational study of 27 recent cases. J. Infect. Dis..

[4] Lanternier F., Sun H.Y., Ribaud P., Singh N., Kontoyiannis D.P., Lortholary O. (2012). Mucormycosis in organ and stem cell transplant recipients. Clin. Infect. Dis..

[5] Lewis R.E., Kontoyiannis D.P. (2013). Epidemiology and treatment of mucormycosis. Future Microbiol..

[6] Chamilos G., Marom E.M., Lewis R.E., Lionakis M.S., Kontoyiannis D.P. (2005). Predictors of pulmonary zygomycosis versus invasive pulmonary aspergillosis in patients with cancer. Clin. Infect. Dis..

[7] Hammond S.P., Baden L.R., Marty F.M. (2011). Mortality in hematologic malignancy and hematopoietic stem cell transplant patients with mucormycosis, 2001 to 2009. Antimicrob. Agents Chemother..

[8] Spellberg B., Edwards J., Ibrahim A. (2005). Novel perspectives on mucormycosis: Pathophysiology, presentation, and management. Clin. Microbiol. Rev..

[9] Caramalho R., Tyndal J.D.A., Monk B.C., Larentis T., Lass-Flörl C., Lackner M. (2017). Intrinsic short-tailed azole resistance in mucormycetes is due to an evolutionary conserved aminoacid substitution of the lanosterol 14α-demethylase. Sci. Rep..

[10] Skiada A., Pagano L., Groll A., Zimmerli S., Dupont B., Lagrou K., Lass-Florl C., Bouza E., Klimko N., Gaustad P. (2011). Zygomycosis in Europe: Analysis of 230 cases accrued by the registry of the European Confederation of Medical Mycology (ECMM) working group on Zygomycosis between 2005 and 2007. Clin. Microbiol. Infect..

[11] Antoniadou A. (2009). Outbreaks of zygomycosis in hospitals. Clin. Microbiol. Infect..

[12] Duffy J., Harris J., Gade L., Sehulster L., Newhouse E., O’Connell H., Noble-Wang J., Rao C., Balajee S.A., Chiller T. (2014). Mucormycosis outbreak associated with hospital linens. Pediatr. Infect. Dis. J..

[13] Garcia-Hermoso D., Criscuolo A., Lee S.C., Legrand M., Chaouat M., Denis B., Lafaurie M., Rouveau M., Soler C., Schaal J.-V. (2018). Outbreak of invasive wound mucormycosis in a burn unit due to multiple strains of *Mucor circinelloides* f. *circinelloides* resolved by whole-genome sequencing. MBio.

[14] Lee S.C., Billmyre R.B., Li A., Carson S., Sykes S.M., Huh E.Y., Mieczkowski P., Ko D.C., Cuomo C.A., Heitman J. (2014). Analysis of a food-borne fungal pathogen outbreak: Virulence and genome of a *Mucor circinelloides* isolate from yogurt. MBio.

[15] Magan N., Olsen M. (2004). Mycotoxins in Food: Detection and Control.

[16] Torres-Martinez S., Ruiz-Vázquez R.M., Garre V., López-García S., Navarro E., Vila A. (2012). Molecular tools for carotenogenesis analysis in the zygomycete *Mucor circinelloides*. Methods Mol. Biol..

[17] Vellanki S., Navarro-Mendoza M.I., Garcia A., Murcia L., Perez-Arques C., Garre V., Nicolas F.E., Lee S.C. (2018). *Mucor circinelloides*: Growth, maintenance, and genetic manipulation. Curr. Protoc. Microbiol..

[18] Nicolas F.E., de Haro J.P., Torres-Martínez S., Ruiz-Vázquez R.M. (2007). Mutants defective in a *Mucor circinelloides dicer*-like gene are not compromised in siRNA silencing but display developmental defects. Fungal Genet. Biol..

[19] Rodriguez-Frometa R.A., Gutiérrez A., Torres-Martínez S., Garre V. (2013). Malic enzyme activity is not the only bottleneck for lipid accumulation in the oleaginous fungus *Mucor circinelloides*. Appl. Microbiol. Biotechnol..

[20] Brock M. (2012). Application of bioluminescence imaging for in vivo monitoring of fungal infections. Int. J. Microbiol..

[21] Delarze E., Ischer F., Sanglard D., Coste A.T. (2015). Adaptation of a *Gaussia princeps* Luciferase reporter system in *Candida albicans* for in vivo detection in the *Galleria mellonella* infection model. Virulence.

[22] Jacobsen I.D., Lüttich A., Kurzai O., Hube B., Brock M. (2014). In vivo imaging of disseminated murine *Candida albicans* infection reveals unexpected host sites of fungal persistence during antifungal therapy. J. Antimicrob. Chemother..

[23] Brock M., Jouvion G., Droin-Bergère S., Dussurget O., Nicola M.-A., Ibrahim-Granet O. (2008). Bioluminescent *Aspergillus fumigatus*, a new tool for drug efficiency testing and in vivo monitoring of invasive aspergillosis. Appl. Environ. Microbiol..

[24] Donat S., Hasenberg M., Schäfer T., Ohlsen K., Gunzer M., Einsele H., Löffler J., Beilhack A., Krappmann S. (2012). Surface display of *Gaussia princeps* luciferase allows sensitive fungal pathogen detection during cutaneous aspergillosis. Virulence.

[25] Ibrahim-Granet O., Jouvion G., Hohl T.M., Droin-Bergère S., Philippart F., Kim O.Y., Adib-Conquy M., Schwendener R., Cavaillon J.M., Brock M. (2010). In vivo bioluminescence imaging and histopathopathologic analysis reveal distinct roles for resident and recruited immune effector cells in defense against invasive aspergillosis. BMC Microbiol..

[26] Slesiona S., Ibrahim-Granet O., Olias P., Brock M., Jacobsen I.D. (2012). Murine infection models for *Aspergillus terreus* pulmonary aspergillosis reveal long-term persistence of conidia and liver degeneration. J. Infect. Dis..

[27] Roncero M.I.G. (1984). Enrichment Method for the isolation of auxotrophic mutants of mucor using the polyene antibiotic *N*-glycosyl-polifungin. Carlsberg Res. Commun..

[28] Nicolas F.E., Navarro-Mendoza M.I., Pérez-Arques C., López-García S., Navarro E., Torres-Martínez S., Garre V. (2018). Molecular tools for carotenogenesis analysis in the mucoral *Mucor circinelloides*. Methods Mol. Biol..

[29] Maurer E., Hörtnagl C., Lackner M., Grässle D., Naschberger V., Moser P., Segal E., Semis M., Lass-Flörl C., Binder U. (2018). *Galleria mellonella* as a model system to study virulence potential of mucormycetes and evaluation of antifungal treatment. Med. Mycol..

[30] Arendrup M.C., Hope W.W., Lass-Flörl C., Cuenca-Estrella M., Arikan S., Barchiesi F., Bille J., Chryssanthou E., Groll A. (2012). EUCAST technical note on the EUCAST definitive document EDef 7.2: Method for the determination of broth dilution minimum inhibitory concentrations of antifungal agents for yeasts EDef 7.2 (EUCAST-AFST). Clin. Microbiol. Infect..

[31] Fallon J., Kelly J., Kavanagh K. (2012). *Galleria mellonella* as a model for fungal pathogenicity testing. Methods Mol. Biol..

[32] Maurer E., Browne N., Surlis C., Jukic E., Moser P., Kavanagh K., Lass-Flörl C., Binder U. (2015). *Galleria mellonella* as a host model to study *Aspergillus terreus* virulence and amphotericin B resistance. Virulence.

[33] Grahl N., Shepardson K.M., Chung D., Cramer R.A. (2012). Hypoxia and fungal pathogenesis: To air or not to air?. Eukaryot. Cell.

[34] Dannaoui E. (2017). Antifungal resistance in mucorales. Int. J. Antimicrob. Agents.

[35] Espinel-Ingroff A., Chakrabarti A., Chowdhary A., Cordoba S., Dannaoui E., Dufresne P., Fothergill A., Ghannoum M., Gonzalez G.M., Guarro J. (2015). Multicenter evaluation of MIC distributions for epidemiologic cutoff value definition to detect amphotericin B, posaconazole, and itraconazole resistance among the most clinically relevant species of Mucorales. Antimicrob. Agents Chemother..

[36] Khan Z.U., Ahmad S., Brazda A., Chandy R. (2009). *Mucor circinelloides* as a cause of invasive maxillofacial zygomycosis: an emerging dimorphic pathogen with reduced susceptibility to posaconazole. J. Clin. Microbiol..

[37] Perkhofer S., Locher M., Cuenca-Estrella M., Rüchel R., Würzner R., Dierich M.P., Lass-Flörl C. (2008). Posaconazole enhances the activity of amphotericin B against hyphae of zygomycetes in vitro. Antimicrob. Agents Chemother..

[38] Salas V., Pastor F.J., Calvo E., Alvarez E., Sutton D.A., Mayayo E., Fothergill A.W., Rinaldi M.G., Guarro J. (2012). In vitro and in vivo activities of posaconazole and amphotericin B in a murine invasive infection by *Mucor circinelloides*: poor efficacy of posaconazole. Antimicrob. Agents Chemother..

[39] Caramalho R., Maurer E., Binder U., Araújo R., Dolatabadi S., Lass-Flörl C., Lackner M. (2015). Etest cannot be recommended for in vitro susceptibility testing of mucorales. Antimicrob. Agents Chemother..

[40] Vitale R.G., de Hoog G.S., Schwarz P., Dannaoui E., Deng S., Machouart M., Voigt K., van de Sande W.W., Dolatabadi S., Meis J.F. (2012). Antifungal susceptibility and phylogeny of opportunistic members of the order mucorales. J. Clin. Microbiol..

[41] Rodriguez M.M., Pastor F.J., Sutton D.A., Calvo E., Fothergill A.W., Salas V., Rinaldi M.G., Guarro J. (2010). Correlation between in vitro activity of posaconazole and in vivo efficacy against *Rhizopus oryzae* infection in mice. Antimicrob. Agents Chemother..

[42] D’Enfert C., Diaquin M., Delit A., Wuscher N., Debeaupuis J.P., Huerre M., Latge J.P. (1996). Attenuated virulence of uridine-uracil auxotrophs of *Aspergillus fumigatus*. Infect. Immun..

[43] Galiger C., Brock M., Jouvion G., Savers A., Parlato M., Ibrahim-Granet O. (2013). Assessment of efficacy of antifungals against *Aspergillus fumigatus*: Value of real-time bioluminescence imaging. Antimicrob. Agents Chemother..

