# Supplementary material for: Generation of A Mucor circinelloides Reporter Strain—A Promising New Tool to Study Antifungal Drug Efficacy and Mucormycosis
Source: Genes (Basel). 2018 Dec 7;9(12):613. doi: 10.3390/genes9120613 (PMC6315630; doi:10.3390/genes9120613)
Supplement: Supplementary file 1 [file genes-09-00613-s001.pdf]

Supplementary Material

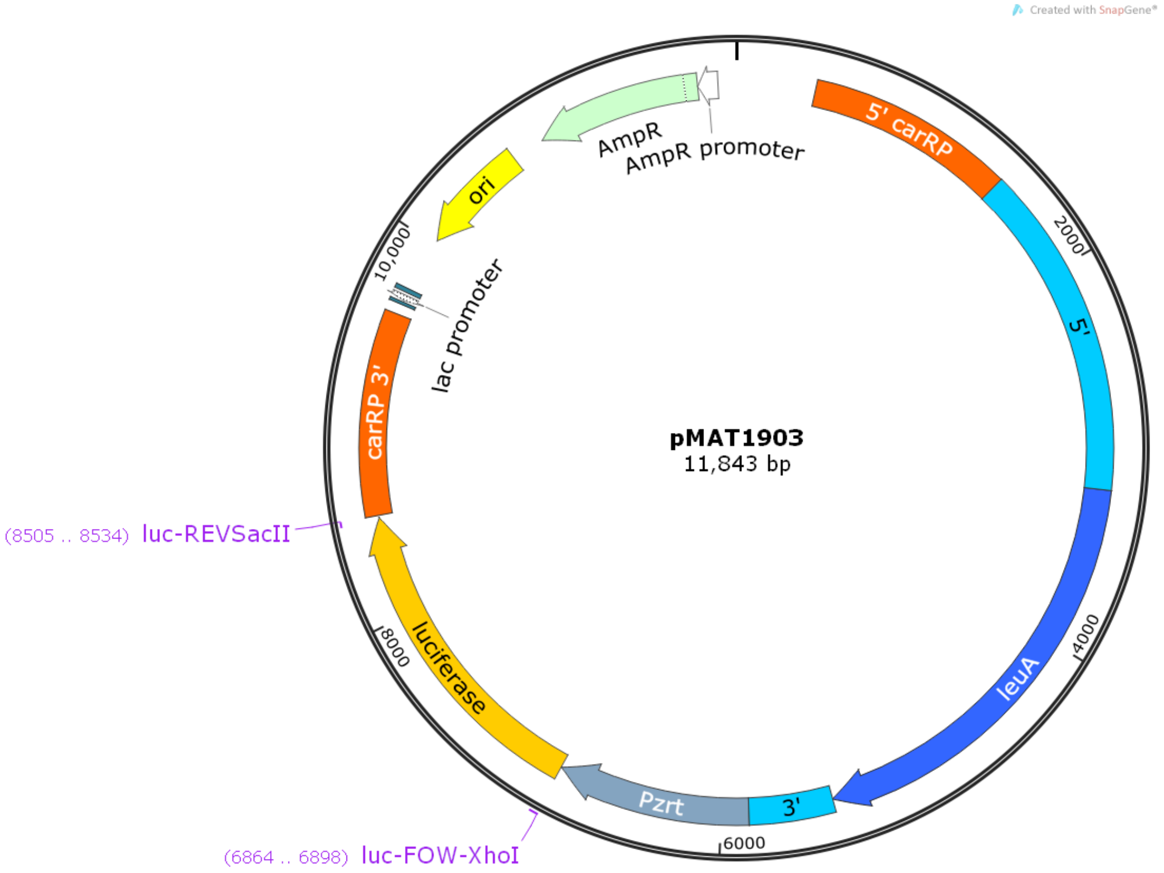

Figure S1: Plasmid pMAT1903 containing luciferase gene without peroxisomal target sequence under the strong *zrt1* promoter and functional *leuA*.

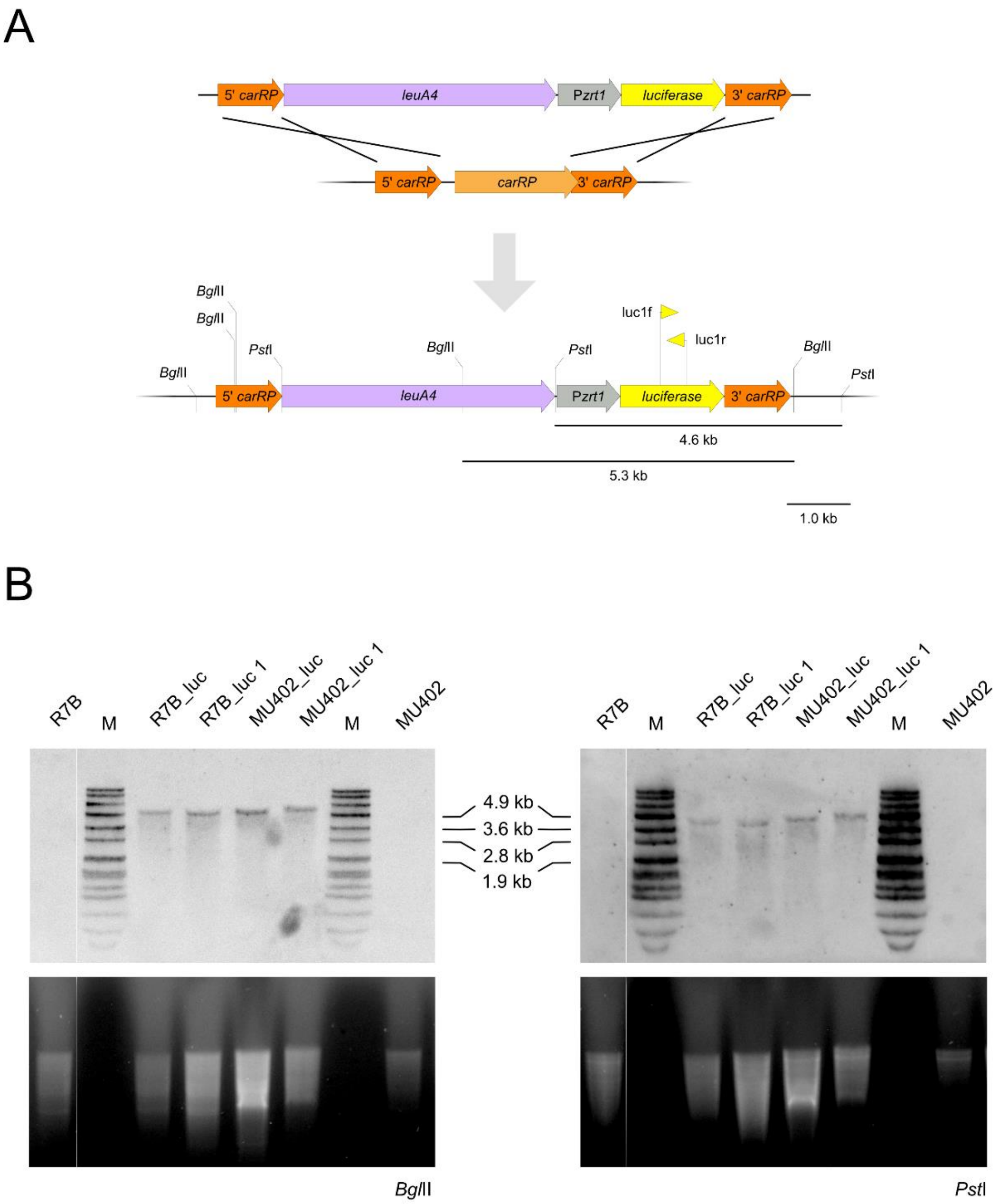

Figure S2: Gene targeting strategy and confirmation of homologous recombination. (A) Schematic presentation of gene targeting and Southern analysis strategy. For generation of the targeting cassette, a leucine auxotrophic marker followed by the gene coding for firefly luciferase were cloned in between 1 kb each of the 5' UTR and the 3' UTR of carRP, which were used as flanks for homologous recombination after linearization of the plasmid by *Cfr*9I (*Xma*I) digestion. Restriction sites (*Bgl*II, *Pst*I) and the site for probe hybridization are shown. Expected fragment length to be identified upon proper integration at the carRP locus are 4.6 kb and 5.3 kb. (B) Southern analysis. Genomic DNA of individual transformants was digested with *Bgl*II and *Pst*I, respectively, separated on a 0.8 % agarose gel (lower

panels) and blotted onto nylon membranes. To determine fragment size, DIG-labelled marker VII (Roche; M) was used; the length of selected marker bands is indicated. Please note that the 3' restriction sites yielding the detected fragments are located outside the sequence provided for homologous recombination and thus are specific for the *carRP* locus.

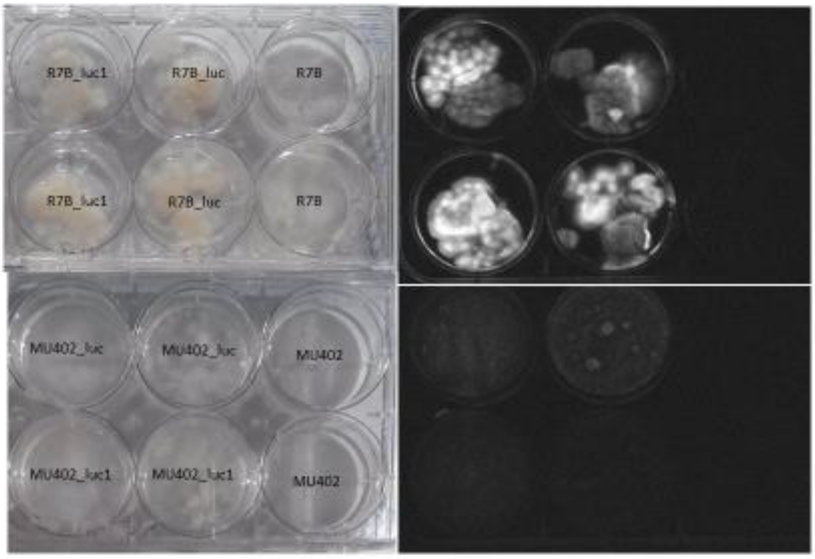

Figure S3: Visualisation of bioluminescence from *M. circinelloides* cultures. The *M. circinelloides* transformants (2 per strain) and the respective parental strains are shown. 10^5 spores/ml were inoculated in YNB medium and grown for 24 h. Light emission was induced by the addition of D-luciferin (10 mM) to the medium, and bioluminescence images of the cultures were acquired by a monochrome scientific grade CCD camera (BIO-VISION 3000 imaging system, right panel).

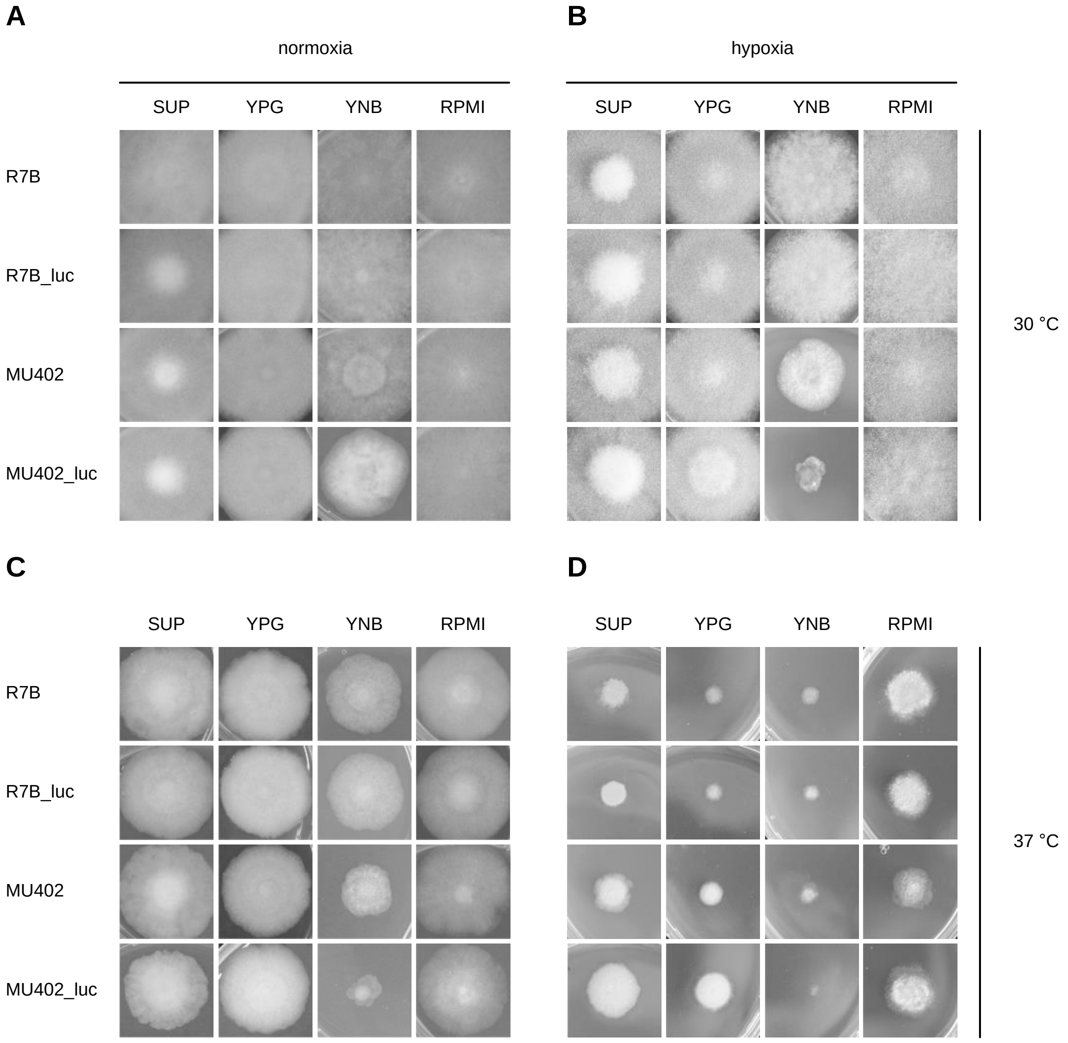

Figure S4: Growth phenotypes of recipient and luciferase expressing strains grown for 48 h on different media at 30 °C (panels A and B) and 37 °C (panels C and D) under normoxic (panels A and C) and hypoxic conditions (panels B and D) are shown. Hypoxia was induced by reducing the oxygen concentration in the incubator to 1 %.

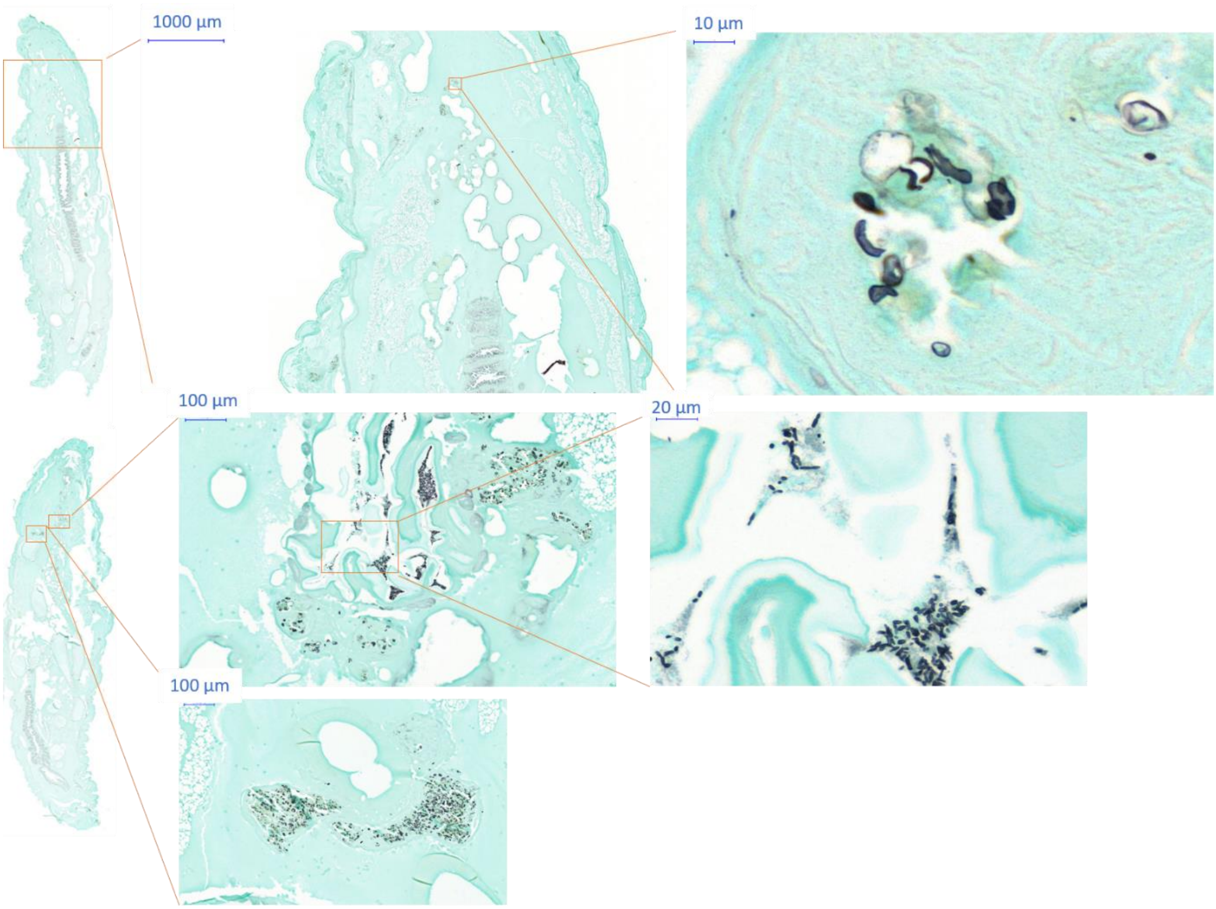

Figure S5: Histological examination of Mucor circinelloides infected Galleria mellonella larvae. Specimen were fixed in formalin 72 h after infection with 10^6 spores of R7B_luc and embedded in paraffin. Tissue sections were prepared at a thickness of 3.0 µm and stained with Grocott silver stain to optimize visualisation of fungal elements.

Table S1: Colony diameter of *M. circinelloides* strains on various growth media. Colony diameter was determined in triplicates after 24 h of incubation at 30 °C and 37 °C. Numbers given represent the average of two independent experiments. Significance was determined by calculating standard deviation (SD).

		**30 °C**		**37 °C**	
**medium**	**strains**	**colony diameter**	**SD**	**colony diameter**	**SD**
**RPMI**	R7B	2,2	0,1	1,2	0,1
	R7B_luc	2,2	0,1	1,4	0,0
	MU402	1,9	0,1	1,2	0,1
	MU402_luc	1,8	0,1	1,1	0,1
**YNB**	R7B	2,1	0,0	1,3	0,1
	R7B_luc	1,9	0,1	1,2	0,0
	MU402	1,2	0,1	0,8	0,0
	MU402_luc	0,8	0,1	0,4	0,0
**YPG**	R7B	2,4	0,0	1,5	0,1
	R7B_luc	2,3	0,1	1,8	0,1
	MU402	2,2	0,0	1,7	0,1
	MU402_luc	2,2	0,0	1,6	0,1
SUP	R7B	2,3	0,0	1,8	0,1
	R7B_luc	2,3	0,1	1,6	0,1
	MU402	2,2	0,1	1,7	0,1
	MU402_luc	2,2	0,0	1,6	0,1
